# Fungal and Bacterial Communities in Indoor Dust Follow Different Environmental Determinants

**DOI:** 10.1371/journal.pone.0154131

**Published:** 2016-04-21

**Authors:** Fabian Weikl, Christina Tischer, Alexander J. Probst, Joachim Heinrich, Iana Markevych, Susanne Jochner, Karin Pritsch

**Affiliations:** 1 Institute of Biochemical Plant Pathology, Helmholtz Zentrum München—German Research Centre for Environmental Health, Neuherberg, Germany; 2 Institute of Epidemiology I, Helmholtz Zentrum München—German Research Centre for Environmental Health, Neuherberg, Germany; 3 Centre for Research in Environmental Epidemiology, Barcelona, Spain; 4 Department of Earth and Planetary Sciences, University of California, Berkeley, California, United States of America; 5 Institute and Outpatient Clinic for Occupational, Social and Environmental Medicine, University Hospital Munich, Ludwig-Maximilians University, Munich, Germany; 6 Division of Metabolic and Nutritional Medicine, Dr. von Hauner Children’s Hospital, Ludwig-Maximilians-University, Munich, Germany; 7 Physical Geography / Landscape Ecology and Sustainable Ecosystem Development, Catholic University of Eichstätt-Ingolstadt, Eichstätt, Germany; Field Museum of Natural History, UNITED STATES

## Abstract

People spend most of their time inside buildings and the indoor microbiome is a major part of our everyday environment. It affects humans’ wellbeing and therefore its composition is important for use in inferring human health impacts. It is still not well understood how environmental conditions affect indoor microbial communities. Existing studies have mostly focussed on the local (e.g., building units) or continental scale and rarely on the regional scale, e.g. a specific metropolitan area. Therefore, we wanted to identify key environmental determinants for the house dust microbiome from an existing collection of spatially (area of Munich, Germany) and temporally (301 days) distributed samples and to determine changes in the community as a function of time. To that end, dust samples that had been collected once from the living room floors of 286 individual households, were profiled for fungal and bacterial community variation and diversity using microbial fingerprinting techniques. The profiles were tested for their association with occupant behaviour, building characteristics, outdoor pollution, vegetation, and urbanization. Our results showed that more environmental and particularly outdoor factors (vegetation, urbanization, airborne particulate matter) affected the community composition of indoor fungi than of bacteria. The passage of time affected fungi and, surprisingly, also strongly affected bacteria. We inferred that fungal communities in indoor dust changed semi-annually, whereas bacterial communities paralleled outdoor plant phenological periods. These differences in temporal dynamics cannot be fully explained and should be further investigated in future studies on indoor microbiomes.

## Introduction

In industrialized countries, people spend a majority of their time indoors, and residential floor space can surpass the land area in a city [1]. Therefore, the building environment can be seen as the “modern ecological habitat of *Homo sapiens sapiens*” with all implications that may have on human well-being [2].

Indoor microbial communities are a ubiquitous part of the building environment. The season has been determined to be significant for fungal composition [3–5], but of minor or no importance for bacteria [6–9]. The outdoor environment has been shown to have a major impact on indoor the fungal community [4, 7, 10], while occupants [7, 11–15] and ventilation types [11, 15] have been found to affect bacteria. However, DNA-based studies with high numbers (> 100) of spatially distributed indoor dust samples mainly focussed on epidemiology (i.e., the human health effects of microbial communities) [16, 17]. Only recently, studies on a continental and global scale revealed that the indoor microbial community depended on the environmental parameters in an individual geographic region, to a minor degree for bacteria, and to a larger degree for fungi [7, 10]. Scales in between continental overviews and local investigations of individual building units have rarely been considered in molecular analyses of indoor dust. Influential factors on a continental scale (e.g., temperature or precipitation [7, 10]) are rather uniform on a regional scale, e.g. in a specific metropolitan area, and factors that affect an individual building may not be significant for the surrounding area. To carve out the reasons for the variation in the indoor fungal and bacterial communities, studies on a regional scale with comprehensive environmental data are required.

The potential effects of the time of the year on the composition of an indoor microbial community have been analysed by sampling at different times or over short periods of time [4, 5, 8]. Continuous observations (i.e., high frequency temporal sampling of indoor dust over several months) have used spore counts or cultivation [3, 18, 19]. In the adjacent outdoor air environment, seasonal changes have been well described in fungi [3, 20–22] and have recently been observed in bacteria during 14 months of monitoring [23], confirming studies that had shorter time-frames [24–26]. In addition, outdoor airborne bacteria can change within few days [27, 28]. Although large knowledge gaps exist, temporal changes in microbial communities seem to be common in a number of other environments as well [29]. For an actual assessment on how microbial communities in indoor dust are affected by the passage of time, comparing different short windows of time is not sufficient. Instead, it is necessary to perform analyses over a period of several months or years with frequent sampling, but such DNA-based studies are rare.

In this study, we determined the variation and diversity of the fungal and bacterial microbiome in dust samples from 286 households. The samples were distributed spatially (i.e., over an area of Munich, Germany) and temporally (i.e., over 10 months). Each household was only sampled once. We used fungal and bacterial fingerprints (terminal restriction fragment length polymorphism–tRFLP [30]) along with associated data sets on environmental parameters.

Our first objective was to identify key indoor and outdoor environmental factors that affected the microbial community. Secondly, we assessed and compared the temporal dynamics of the fungal and dust bacterial communities. Regarding that, we explored whether the existing sample design of spatially unrelated single samples collected in a defined time window (each home sampled once) could be used to infer the temporal dynamics for fungi and bacteria. In addition, we showed that the seasonal effects on indoor communities can be different for fungi and bacteria.

## Materials and Methods

### Study design and sampling

We analysed microbial fingerprints derived from DNA extracts of dust from the living room floors of 286 homes. Each home was sampled once within 301 days from April 1998 to February 1999. All dust samples were collected in an urban area in Munich (radius: 37.5 km, S1 File) as part of the LISAplus study (i.e., The influence of life-style factors on the development of the immune system and allergies in East and West Germany PLUS the influence of traffic emissions and genetics study). LISAplus is an ongoing birth cohort study that has screening, recruitment and exclusion criteria that have been described elsewhere [31, 32]. LISAplus has been approved by local ethics committees (Ethikkommission der Bayrischen Landesärztekammer, Ethikkommission an der Medizinischen Fakultät der Universität Leipzig, Ärztekammer Nordrhein) and written informed consent was obtained from all participating families.

Information on indoor and most outdoor environmental characteristics for the sampled homes was obtained by self-completed questionnaires. Based on the residential addresses, we acquired further information on air pollution from traffic, the surrounding greenness based on satellite-data (i.e., vegetation density, the Normalized Difference Vegetation Index (NDVI)) and the urban index (i.e., the proportion of the built-up area). A detailed description of the environmental characteristics is given in S2 File.

Samples were collected by trained inspectors using vacuum cleaners (Phillips, Hamburg, Germany) equipped with ALK filter holders (ALK, Hørsholm, Denmark) containing a paper filter when the child of a family that participated in the cohort study was two to three months old. The sampling was done by vacuuming 1 m^2^ for two minutes (textile floors) or 4 m^2^ for four minutes (smooth floors). The filter boxes were stored below −20°C. A detailed description of the dust sampling and processing has been previously published [31, 33].

### Microbial fingerprinting

Frozen filter boxes with vacuumed dust were equilibrated to ambient conditions for 60 minutes in a clean PCR chamber (airflow deactivated). Dust was released from the filter boxes, freed from obvious extraction obstacles (e.g., stones, etc.) and 100 mg were used for DNA extraction with a PowerSoil-htp96 Soil DNA Isolation Kit (Mo-Bio Laboratories, Carlsbad, CA, USA). Contamination was controlled with samples consisting of small pieces of filter material from empty dust-filters, and with negative controls during PCR. For tRFLP DNA-fingerprinting, the DNA was PCR-amplified using a TopTaq DNA polymerase kit (Qiagen, Hilden, Germany) with the primers ITS1F (5’-CTTGGTCATTTAGAGGAAGTAA-3’) [34] and ITS4 (5’-TCCTCCGCTTATTGATATGC-3’) [35] for fungal ITS (internal transcribed spacer) DNA, or Bac27f (5’-AGAGTTTGATCMTGGCTCAG-3’) [36] and 907r (5-CCGTCAATTCMTTTGAGTTT-3) [37] for bacterial 16S rRNA genes. Forward primers were labelled with 6-FAM and reverse primers with 6-HEX fluorescent dyes, respectively. The PCR profiles were [4 min 94°C; 32 cycles of 60 s 94°C, 60 s 50°C, 90 s 72°C; 5 min 72°C] (fungi) and [5 min 94°C; 30 cycles of 45 s 94°C, 45 s 59°C, 45 s 72°C; 5 min 72°C] (bacteria). Products from two PCR reactions were pooled, purified and digested with the restriction enzyme HpyCH4IV (fungi) or MspI (bacteria). HpyCH4IV was selected after in silico enzyme digestions using REPK v1.3 [38] against an artificial set of fungal sequences commonly found in dust. Cleaned fragments were transferred to HiDi Formamide (Applied Biosystems, Foster City, CA, USA) containing MapMarker 1000-ROX (1:400; Bioventures, Murfreesboro, TN, USA) and separated on an ABI 3730 capillary sequencer (Applied Biosystems). Raw fragment tables were built with peak-scanner 2.0 (Applied Biosystems). T-REX v1.14 [39] was used for noise filtering (peak height, multiplier 1) and binning of fragments (threshold 1 bp).

### Statistical analyses

The R programming environment [40] was used for all statistical analyses. Bray—Curtis dissimilarities [41] between the samples (community variation) and biodiversity indices were calculated from 10^3^ times randomly rarefied (to the lowest amount of signal present in one sample, i.e. between 1677 fluorescence units for forward fragments from bacteria and 3448 fluorescence units for reverse fragments from fungi) OTU (operational taxonomic unit) abundances using algorithms from the vegan [42] and GUniFrac [43] packages. The results from forward and reverse terminal restriction fragments (including labelled forward or reverse primers) were averaged. Multivariate testing for the effect of environmental characteristics on the community was conducted using average Bray—Curtis dissimilarity matrices with the Adonis (Permutational Multivariate Analysis of Variance Using Distance Matrices) and MRPP (Multi-Response Permutation Procedure) functions, each with 10^5^ permutations available in the vegan package. Partial Mantel tests (10^4^ permutations, implemented in the vegan package) were used to test for possible geographic correlations of community dissimilarities.

Microbial diversity was assessed with the Simpson (1–D) and Shannon (H’) indices, and seasonal influences were additionally evaluated with Pielou’s Evenness (J) and Richness (S). Details on these calculations are available in the vegan package [42]. Relationships between biodiversity indices and environmental characteristics were assessed using a Wilcoxon signed rank test for dichotomous variables and a Kruskall—Wallis test for variables with three or more categories due to the non-normal distribution of fungal and bacterial diversity (Shapiro—Wilk test *P* < 0.01).

## Results

### Community variation

The variation in the fungal community was more sensitive to the tested environmental determinants than the variation in the bacterial community (Table 1).

**Table 1:** Significance of associations between environmental determinants and microbial community variation (based on Bray—Curtis dissimilarities).

	Fungi		Bacteria	
Environmental characteristics	*P* ^*a*^	*δ* ^*b*^	*P* ^c^	*δ* ^*d*^
**Indoor characteristics**				
N° of rooms within the flat	0.75	0.87	0.71	0.79
N° of occupants in the flat	0.85	0.79	0.36	0.28
Dampness	0.69	0.29	0.16	0.39
Mould at home	**0.04**	**0.03**	0.09	0.13
Water leakage	0.81	0.85	0.57	0.62
Tightness of the windows ^e^	**0.03**	**0.04**	0.36	0.36
Ventilation living room through windows—summer	0.27	0.24	0.71	0.93
Ventilation living room through windows—winter	0.67	0.64	**0.05**	**0.05**
Heating within the home	**0.03**	**0.02**	0.36	0.41
Renovation measures last 12 months	0.44	0.61	0.65	0.65
Pets	0.27	0.28	0.62	0.75
Type of living room floor	**< 0.001**	**< 0.001**	0.08	**0.02**
Smoking of tobacco in the flat	0.42	0.41	0.71	0.78
**Outdoor characteristics**				
Age of the building	**0.01**	**0.01**	0.28	0.31
Position of the home	0.49	0.67	0.1	**0.05**
Building density of the neighbourhood	0.52	0.59	0.39	0.44
Traffic jams in rush hour	0.83	0.83	0.29	0.24
Facility with noticeable air pollution within 50 and 100 m	0.58	0.72	0.45	0.40
Facility with noticeable air pollution within 50 m	0.33	0.28	0.85	0.94
Surrounding greenness (500 m buffer)	0.72	0.63	084	0.94
Surrounding greenness (100 m buffer)	**0.05**	**0.006**	0.19	0.22
Surrounding greenness (30 m buffer)	0.06	**0.01**	0.33	0.30
Urban index	**0.02**	**0.01**	0.51	0.60
NO_2_	0.23	0.06	0.63	0.75
NO_x_	0.06	**0.03**	0.37	0.41
PM_2.5_	**0.004**	**0.005**	0.51	0.44
PM_10_	0.54	0.32	0.82	0.70
PM_coarse_	**0.04**	**0.008**	0.41	0.46
PM _**2.5**_ absorbance	0.07	0.06	0.37	0.42

Results from Adonis (*P*) and MRPP (*δ*), bold: *P* or *δ* ≤ 0.05; all *R*^*2*^ (Adonis) *A* (MRPP chance corrected between groups agreement) values are given in S1 Table.

^a^ maximum *R*^*2*^ in this column: 0.06

^b^ maximum *A* in this column: 0.03

^c^ maximum *R*^*2*^ in this column: 0.02

^d^ maximum *A* in this column: 0.01

^e^ windows closing with a big or small air gap.

For indoor factors, variation in the fungal community in the living room floor dust was affected in particular by signs of mould inside the home, the tightness of the windows (closing with a big or small air gap), heating inside the home, and the type of living room floor (i.e., Adonis *P* < 0.05, MRPP *δ* < 0.05; Table 1). In contrast, the bacterial community variation was only significantly affected by ventilation (winter half year, Adonis *P* = 0.05, MRPP *δ* = 0.05), and potentially (i.e., Adonis *P* or MRPP *δ* > 0.05 but not both tests) by the type of living room floor (Adonis *P* = 0.08, MRPP *δ* = 0.02) as well as the position of the home (Adonis *P* = 0.1, MRPP *δ* = 0.05).

For outdoor factors (Table 1), a significant effect on the variation in the fungal community was due to the building age, the surrounding greenery within a 100 m buffer, the urbanization grade (urban index), and particulate matter (< 2.5 μm and coarse particulates) (Adonis *P* ≤ 0.05, MRPP *δ* < 0.05). The surrounding greenery within a 30 m buffer (Adonis *P* = 0.06, MRPP *δ* = 0.03) and nitrogen oxides (NO_x_) (Adonis *P* = 0.06, MRPP *δ* = 0.01) were also potentially associated with the variation in the fungal community. The only outdoor characteristic that may have affected variation in the bacterial community was the position of the home (i.e., the level above ground) (Adonis *P* = 0.1, MRPP *δ* = 0.05). No spatial correlation with the community variation was observed (simulated *P* > 0.25 with 10^5^ replicates for fungi and bacteria in partial Mantel tests conditioned by sampling date).

The fungal and bacterial communities showed a significant change with the time of the year (Table 2); its ecological relevance (MRPP *A*, chance-corrected within group agreement) was greater than that of all other tested variables (Table 2 and S1 Table). Of all seasonal categorizations, the outdoor plant phenological periods which reflect the growth stage of vegetation best described the association between the sampling date and the microbial community (fungi: Adonis *R*^2^ = 0.19, MRPP *A* = 0.11; bacteria: *R*^2^ = 0.34, *A* = 0.21) (Table 2).

**Table 2:** Significance of associations between sampling time and microbial parameters.

	representation of sampling times	Community variation				Diversity indices			
		Adonis		MRPP		*P*-values of Kruskal–Wallis tests			
		*P*	*R*^*2*^	*δ*	*A*	Shannon H’	Simpson 1−D	Evenness J	Richness S
**fungi**	**four astronomical seasons** ^**a**^	<10^−5^	0.131	<10^−5^	0.076	0.0018	0.0113	0.0553	0.0001
***bacteria***		*<10*^*−5*^	*0*.*245*	*<10*^*−5*^	*0*.*151*	*<10*^*−5*^	*<10*^*−5*^	*<10*^*−5*^	*<10*^*−5*^
**fungi**	**four meteorological seasons** ^**b**^	<10^−5^	0.145	<10^−5^	0.085	0.0003	0.0002	0.0006	0.0003
***bacteria***		*<10*^*−5*^	*0*.*248*	*<10*^*−5*^	*0*.*156*	*<10*^*−5*^	*<10*^*−5*^	*<10*^*−5*^	*<10*^*−5*^
**fungi**	**nine phenological periods** ^**c**^	<10^−5^	0.187	<10^−5^	0.111	<10^−5^	<10^−5^	10^−5^	10^−5^
***bacteria***		*<10*^*−5*^	*0*.*341*	*<10*^*−5*^	*0*.*205*	*<10*^*−5*^	*<10*^*−5*^	*<10*^*−5*^	*<10*^*−5*^
**fungi**	**continuous (metric)**	0.0267	0.013						
***bacteria***		*<10*^*−5*^	*0*.*228*						

Categorization of the sampling time and its significance for differences in community variation (based on Bray—Curtis dissimilarities) and diversity changes for fungi and bacteria (Shannon Index (H’), Simpson Index (1-D), Pielou’s Evenness (J), Richness (S)).

^a^ e.g., spring starting at March equinox, ending at June solstice

^b^ e.g., spring starting March 1, ending May 31

**Fig 1:**
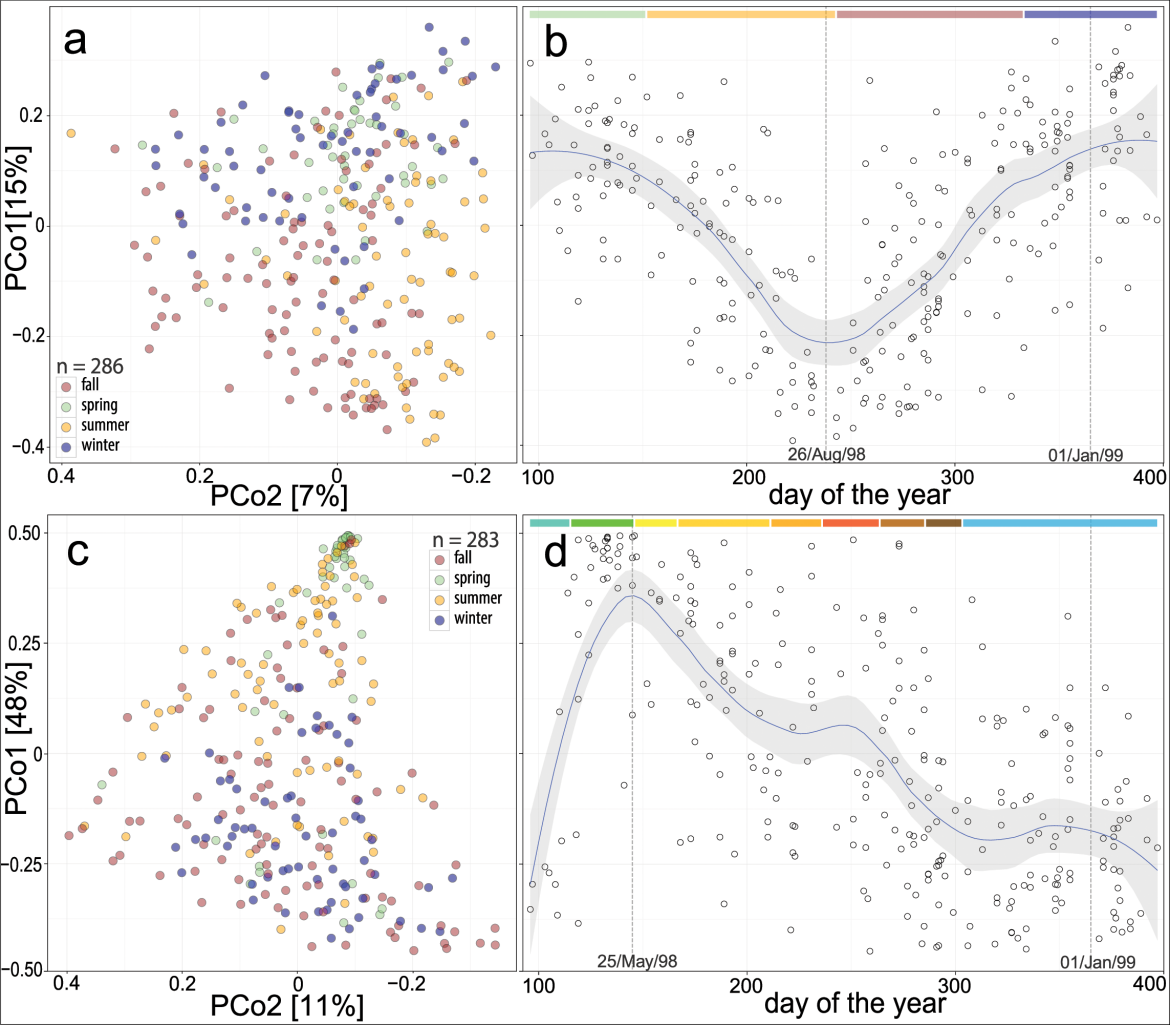
Microbial community variation and temporal dynamics. Points: dust samples from different homes. **(a, c)** first coordinates of a principal coordinate analysis (PCoA) on Bray–Curtis dissimilarities of the fungal (a) and bacterial (c) communities; in brackets: explained variance, colours: meteorological seasons. **(b, d)** the principal coordinate that explains most of the variation for fungi (b) or bacteria (d) sorted by the sampling date; regression (b, d): a locally weighted polynomial fit with 95% confidence interval; coloured bars at the top of (b): meteorological seasons: spring, summer, autumn, and winter; coloured bars at the top of (d): plant phenological periods of the geographic area during the time-frame of sampling: early spring (beginning March 16, 1998), full spring (April 25), early summer (May 26), midsummer (June 16), late summer (August 1), early autumn (August 26), full autumn (September 23), late autumn (October 15), and winter (November 3) (dates obtained from: http://www.dwd.de/, http://www.phenocal.chira.de; accessed 18 Jul 2014).

^c^ for the area of Munich in 1998−1999, displayed and supported in Fig 1D.

Chronological presentations of the first Principal Coordinates (PCo) in a PCo analysis (PCoA) showed different time courses of the community variation for fungi and bacteria (considering all samples, Fig 1). The PCoA indicated that fungal community variation changed semi-annually; it gradually changed from winter to summer and vice versa (for the 1^st^ PCo in Fig 1B: approximately 5% of the total variation between samples occurred during the first 5 months). In contrast, the bacterial community variation changed rapidly and extensively during the phenological periods of early and full spring (for the 1^st^ PCo in Fig 1D: approximately 35% of the total variation between samples occurred during the first 2 months) and gradually returned to a winter state until the beginning of phenological winter.

### Diversity

The determinants of significantly different community variations in fungi and bacteria were not associated per se with significantly different diversities (Simpson and Shannon indices, Table 3: significant variables, S2 Table: all variables).

**Table 3:** Significant associations between environmental determinants and microbial diversity.

Environmental characteristics	Fungi 1-D median (p25-p75)	*P*	Fungi H’ median (p25-p75)	*P*	Bacteria 1-D median (p25-p75)	*P*	Bacteria H’Median (p25-p75)	*P*
N° of occupants in the flat		0.88		0.80		**0.03**		**0.01**
2–3 persons	0.90(0.84–0.94)		3.20(2.81–3.61)		0.77(0.58–0.85)		2.14(1.59–2.63)	
4 persons	0.91(0.84–0.94)		3.38(2.88–3.65)		0.83(0.69–0.88)		2.52(1.94–2.84)	
5–6 persons	0.91(0.85–0.94)		3.39(2.9–3.67)		0.80(0.54–0.87)		2.36(1.40–2.82)	
Vent. living room: summer		0.08		**0.04**		0.62		0.56
seldom/never/via another room	0.91(0.85–0.93)		3.16(2.79–3.47)		0.71(0.53–0.86)		2.00(1.47–2.57)	
once/several times a day (short)	0.93(0.88–0.94)		3.54(3.04–3.72)		0.77(0.52–0.86)		2.21(1.36–2.66)	
once/several times a day (long)	0.90(0.83–0.94)		3.20(2.78–3.6)		0.79(0.62–0.86)		2.26(1.6–2.71)	
Type of living room floor		0.30		0.33		**0.04**		**0.01**
carpet	0.92(0.86–0.94)		3.35(2.95–3.64)		0.78(0.51–0.85)		2.14(1.35–2.58)	
smooth	0.90(0.81–0.94)		3.09(2.78–3.6)		0.81(0.58–0.87)		2.32(1.46–2.71)	
smooth with rugs	0.90(0.83–0.93)		3.20(2.74–3.61)		0.80(0.68–0.87)		2.36(1.84–2.81)	
Position of the home		0.15		0.09		**0.04**		**0.03**
ground floor	0.91(0.84–0.94)		3.3(2.81–3.66)		0.81(0.64–0.88)		2.35(1.73–2.8)	
1^st^ floor	0.92(0.85–0.95)		3.45(2.93–3.67)		0.83(0.63–0.86)		2.41(1.71–2.74)	
2^nd^ floor	0.89(0.83–0.92)		3.06(2.74–3.43)		0.78(0.57–0.82)		2.21(1.62–2.37)	
3^rd^ floor or higher	0.89(0.82–0.93)		3.1(2.72–3.58)		0.75(0.45–0.85)		2.12(1.25–2.64)	
Surrounding greenness (100 m buffer)		**0.03**		0.11		0.14		0.11
1^st^ tertile (0.06–0.26)	0.92(0.86–0.94)		3.40(2.92–3.65)		0.79(0.63–0.86)		2.22(1.63–2.68)	
2^nd^ tertile (0.27–0.33)	0.88(0.81–0.93)		3.10(2.65–3.59)		0.75(0.54–0.85)		2.14(1.44–2.65)	
3^rd^ tertile (0.33–0.59)	0.90(0.84–0.94)		3.27(2.85–3.67)		0.81(0.69–0.87)		2.42(1.84–2.74)	

Variables with significant (Wilcoxon signed rank test or Kruskall–Wallis test *P* < 0.05) associations to diversity changes (Simpson Index (1-D), Shannon Index (H’)) for fungi and bacteria. p25-p75: interquartile ranges; bold: *P* ≤ 0.05. Values for all insignificant (*P* > 0.05) variables are given in S2 Table.

For indoor environmental factors, infrequent ventilation of a flat via the windows in the summer half year was significantly associated with a higher diversity of less abundant fungi (Shannon index *P* = 0.04, Kruskal—Wallis test). Households with more than three occupants had a higher bacterial diversity than smaller households (Shannon index *P* = 0.01 and Simpson index *P* = 0.03, Kruskal—Wallis tests). Homes with carpets had a lower bacterial diversity than homes with smooth floors, but the highest bacterial diversity was sampled from mixed floors (Shannon index *P* = 0.01 and Simpson index *P* = 0.04, Kruskal—Wallis tests).

Regarding outdoor factors, low and high (1^st^ and 3^rd^ tertile) surrounding greenery within a 100 m buffer significantly increased the fungal diversity in the dust compared to the third of samples from homes with a medium level of surrounding greenery (2^nd^ tertile) (Simpson index *P* = 0.03, Kruskal—Wallis test). The bacterial diversity was significantly lower for homes above the 1^st^ floor (Shannon index *P* = 0.04 and Simpson index *P* = 0.03, Kruskal—Wallis tests).

**Fig 2:**
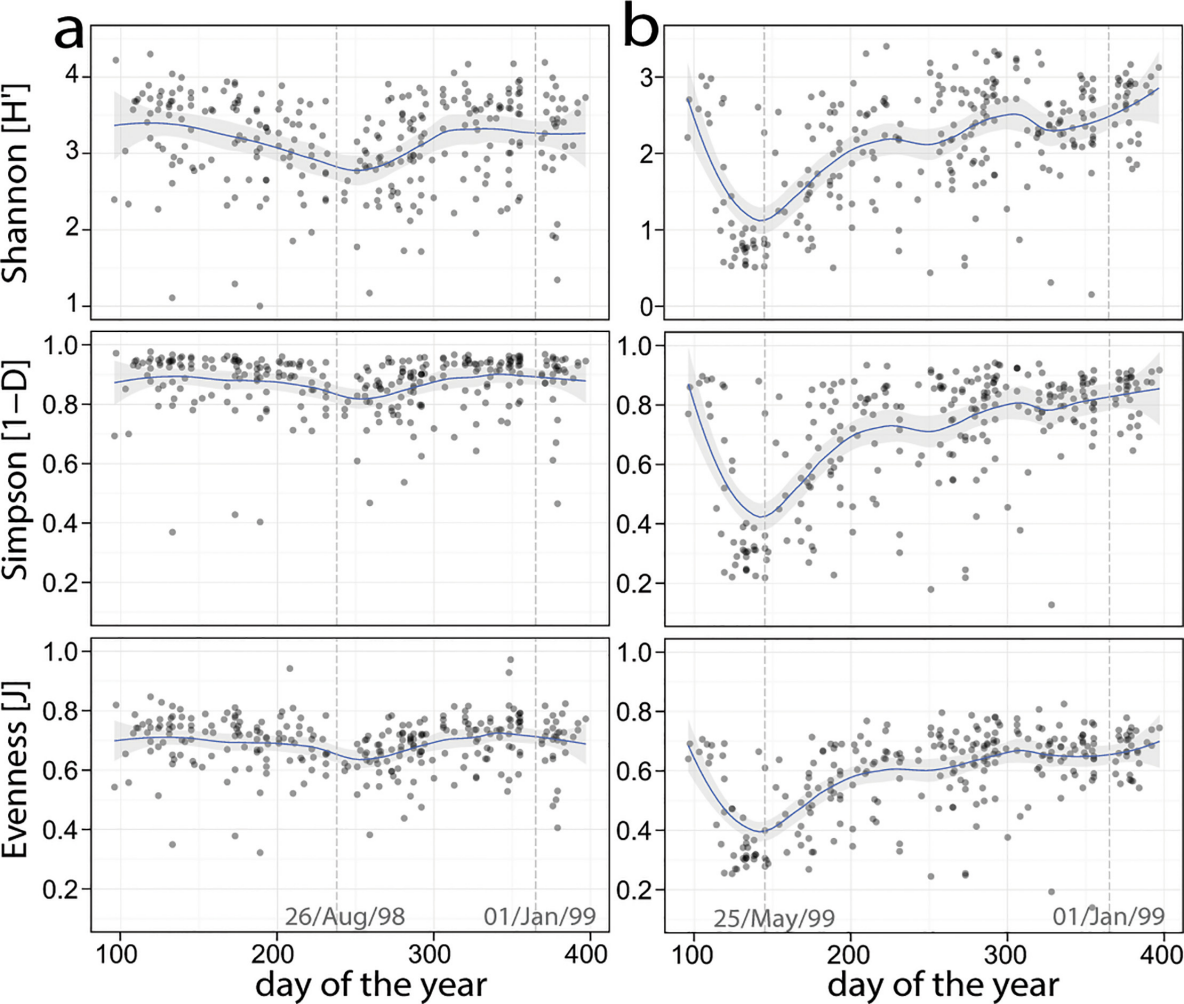
Temporal dynamics of microbial diversity. Points: dust samples from different homes. Diversity indices of the dust samples are sorted by sampling date, (a) for fungi (n = 286) and (b) for bacteria (n = 283). Regression: a locally weighted polynomial fit with 95% confidence interval.

Fungal diversity was only lower in late summer compared to the rest of the year (Fig 2A). However, the effect of the season on fungal diversity indices was strongly significant when the sampling times were categorized according to outdoor plant phenology or the meteorological seasons (*P* < 0.001, Kruskal—Wallis tests on Simpson and Shannon indices, Evenness, and Richness; Table 2). In contrast, the bacterial diversity precisely mirrored the shift in the bacterial community variation during spring (Fig 2B). Differences between the maximum and minimum diversity (determined from the localized regression shown in Fig 2) were also much less for fungi (approximate difference: Shannon index 20%, Simpson index 11%, evenness 11%) than for bacteria (approximate difference: Shannon index 61%, Simpson index 52%, evenness 42%).

## Discussion

This study considered both the fungal and the bacterial microbiomes in indoor dust, in more than 250 households in a metropolitan area. We observed that the environmental factors that affected the composition of the fungal microbiome were different than those that affected the bacterial microbiome. However, the time of the year (season or plant phenological period) was the most decisive of the parameters tested for both fungi and bacteria.

Fungal community variation and diversity were influenced by several indoor environmental factors. In contrast, bacterial community variation was significantly affected only by behaviour regarding ventilation, and bacterial diversity was significantly affected only by the number of occupants and the type of living room floor. Similar results have been recently reported [6, 7], and our findings are in accord with studies on the building environment microbiome, which emphasize the importance of the architectural design (including ventilation), occupancy, and human behaviour itself as decisive factors for the biogeography of bacterial communities in indoor environments [7, 11, 15]. In a recent study [6], the fungal community composition in settled dust samples from various indoor locations seemed to be mainly driven by fungi associated with the outdoor environment, while for the bacterial community, the composition reflected the taxa released from the residents, an observation that had also been made earlier [13]. In the same study [6], the authors did not detect any effect of the house or residential characteristics on the bacterial microbiome, which is in accord with our study’s results on community variation. However, we found an effect of two determinants that were kept uniform in their study [6]. The ventilation behaviour showed an effect on the bacterial community variation, as was also previously reported [15]. Additionally, the type of living room floor affected the bacterial diversity. It is apparent that small-scale structured floors (i.e., smooth with rugs) can harbour a higher diversity than homogenous surfaces.

Fungal community characteristics were not affected by dampness or water leakage but were affected by the presence of mould. In a study comparing 17 homes with low mouldiness (evaluated with “Environmental Relative Moldiness Index”, [44]) with 18 homes that had high mouldiness, fungal Richness was significantly associated with the relative humidity but not Evenness or the Shannon or Simpson indices [45]. These findings suggest that dampness alone might be a relatively weak determinant of the indoor fungal community, notwithstanding that the growth of mould is often associated with dampness [46] because different individual sites have different reactions to moisture, and the assessment of dampness and the moisture status is complex [47].

The ventilation strategy in a university building has been identified as one of the strongest factors affecting bacterial community variation [11]. In this study, the association between ventilation habits and bacterial community variation was also significant, but its likely impact (MRPP *A*) was more than 10-fold smaller than the impact of the outdoor phenology (i.e., the sampling time). The probable reason for this difference may be the different environments sampled in the study of Meadow et al. (2014) [11] and the present work: air sampling versus floor dust, mechanic ventilation versus non-mechanic ventilation through windows, etc. In this study, tight windows (which implied a lower air exchange rate) were also significantly associated with fungal community variation and higher fungal diversity.

In accordance to our study, previous work [6] did not find a significantly changing bacterial community variation with rising numbers of residents. However, recently the number of inhabitants was found weakly predictive for the relative abundance of bacteria that were preferentially found indoors [7]. In a similar manner, a higher number of occupants was associated with a higher bacterial diversity in the homes investigated in our study.

Along with building structures and residential characteristics, the presence of pets (i.e. cats and dogs) has also been found to influence the house-associated microbial community [7, 48, 49]. Differently, in our study, the presence of pets at the time of dust sampling did not show a significant effect on the fungal or on the bacterial microbiome. However, previous studies sampled different indoor surfaces [7, 48, 49] and included relatively more pet-owning households [48, 49], while in this study 83% of the homes were entirely devoid of pets and for example only 4% owned dogs. This suggests that effects pets could have on the floor dust microbiome could have been concealed by the variation within our large sample of pet-free households. A greater depth of analysis of sequencing approaches might allow carving out significant differences in bacterial genera between pet and no-pet households, as for example in a study that sampled dust from door rims [7].

The outdoor environment decisively influences the indoor environment with residential and building characteristics mediating the association [6, 13, 50]. In this context, various types of land use have been found to significantly shape the bacterial signature in outdoor air [51, 52]. In our urban environment, we could not confirm that the closer neighbourhood (< 500 m, Table 1 “Outdoor characteristics”) is directly associated with the indoor bacterial community. However, we observed an association with the position of the home. It can be speculated that fewer bacteria are carried from outdoors by the occupants into the flats at a higher level above ground. However, it is also possible that the direct influx of airborne bacteria through the windows varies with the elevation above ground, although such a relationship has been only been found for cultured bacteria in a study that investigated a large elevation difference (238 m) [53]. Future work is necessary to confirm this result by accounting for the building height.

We observed an association of the fungal community with the greenery surrounding the homes, the grade of urbanization and level of airborne particulate matter. Correlations between particulate matter and airborne fungi were previously observed [54], and the fungi themselves may make up a significant fraction of the airborne particulate matter [55, 56]. Additionally, plants are a major source of airborne fungi [57], which may explain the influence of the surrounding greenery we observed for the fungi in indoor dust. In a study on a continental scale in the USA, urbanization was not generally associated with changes in the microbial community of external household surfaces compared to rural areas; however, it tended to lead to a more homogenous community composition [58]. Nevertheless, the number of comparable investigations involving urbanization, exhaust, and greenery is limited, and further studies are necessary in order to confirm their impact on the composition of the fungal microbiome of indoor dust. The results from such studies might identify key environmental characteristics concerning the closer neighbourhood with the potential to create surroundings that promote healthy living [59].

The microbial community structure followed a temporal pattern, which was the major factor affecting the variables considered for fungal and bacterial communities in living room dust. For fungi, semi-annual patterns in their quantity in the indoor environment have previously been repeatedly found by spore counts and cultivation, which were correlated with outdoor concentrations [3]. Additionally, molecular studies on smaller time windows suggested such patterns in different geographical regions [4, 5]. For bacteria, the influence of the sampling time was determined to be not very important in earlier studies [6–9]. Our finding that bacterial communities are considerably influenced by seasons is probably because of the large number of individual samples that were analysed in this study. Almost daily sampling allowed the delineation of temporal dynamics, overcoming inevitable individual variation between locations. A recent investigation on one housing complex with 11 units sampled during one month in summer and in winter found a strong seasonality of the indoor fungal community but little evidence for the same in the bacterial community [6]. However, the authors presumed that a seasonal relationship for bacteria was obscured in their study because of large amounts of human-associated bacteria in the dust samples analysed. Additionally, studies that compare different time windows during the year might also overlook bacterial indoor seasonality. For example, using our data, a comparison of all of the July-August samples with the December-January samples would have underestimated the total annual influence of seasonal change, and for a period of approximately 100 days during phenological winter we would have found almost no effect of this parameter at all (cf. Figs 1 and 2).

The above mentioned seasonal patterns of community change were dissimilar for fungi and bacteria. However, the varying taxonomic resolution of the fingerprinting method restricts an explanation of the relationship between changes in community variation and diversity to a general level. For fungi, a decrease in diversity during summer suggests an influx of high amounts of a few OTUs, such as *Alternaria* and *Cladosporium* [21, 22]. For bacteria, the rapid and strong change in community variation and reduced diversity during the spring suggest a substantial and rapid influx of interrelated OTUs during the early plant flowering (i.e., the full spring phenological period).

In this study, the indoor bacterial community variation and the outdoor plant phenological period in which the samples were taken were strongly associated. However, the fingerprinting technique used did not allow the identification of microbial taxa, and so we could not explore whether these shifts were caused by plant-related taxa. Recently, sources for bacteria in particulate matter of outdoor aerosols from Colorado, USA, were tracked to leaves, soils and cow faeces [25]. In Milan, Italy, plant-derived microbes dominated outdoor airborne bacteria during the summer, while spore-forming bacteria dominated in the winter [26]. Outdoor airborne bacteria sampled during the spring in northern France were also mainly plant-derived [60]. All of this evidence suggests that plant phenology was indeed the cause for the association we found between a change in the bacterial indoor dust community and the full spring period.

## Conclusions

We confirmed earlier findings that the fungal microbiome in indoor dust is more strongly affected than the bacterial microbiome by both, indoor and especially outdoor factors on the scale of an urban metropolitan area, and by using a multitude of locations that were sampled once but time shifted.

Samples from studies similar to ours, i.e., studies that had independent sampling locations on a regional scale and a time-frame of several months, could be used to confirm the unexpectedly strong effect on indoor bacteria that we found for the sampling time during the year.

The semi-annual cycle for the fungal indoor community that we inferred from our samples is similar to the well-explained seasonal change in indoor and outdoor fungal propagules. In contrast, changes in indoor bacteria must be elucidated in future studies. The local plant phenology, particularly at the annual onset of the flowering period, may well be a major driver of temporal change in the indoor bacterial microbiome in many geographical regions.

## Supporting Information

S1 File Distribution of samples. (DOCX)

S2 File Environmental characteristics. (DOCX)

S1 Table Community variation. (DOCX)

S2 Table Significance of associations with diversity–all variables. (DOCX)
