# Supplementary material for: Fungal and Bacterial Communities in Indoor Dust Follow Different Environmental Determinants
Source: PLoS One. 2016 Apr 21;11(4):e0154131. doi: 10.1371/journal.pone.0154131 (PMC4839684; doi:10.1371/journal.pone.0154131)
Supplement: S1 File — (DOCX) [file pone.0154131.s001.docx]

S1 File. Distribution of samples in the Munich urban area.

The sampling scheme for house dusts can be considered as random single time-point sampling, which was originally done for a population based cohort study targeting newborns in urban environments (1). Hereby, the random factor was defined by the date of birth of a child in an obstetrical clinic in Munich (2) and the location of the households in which the newborns lived until the date of dust sampling, which was in the third month after birth. The map below illustrates the result of this random sampling by showing the distribution of all sampling locations and seasons across the Munich metropolitan area. It covers the biggest part of the ‘Larger Urban Zone’ (3) of Munich and includes the position of 280 sampled households located within a radius of 35 km from the center of Munich (98% of all samples).


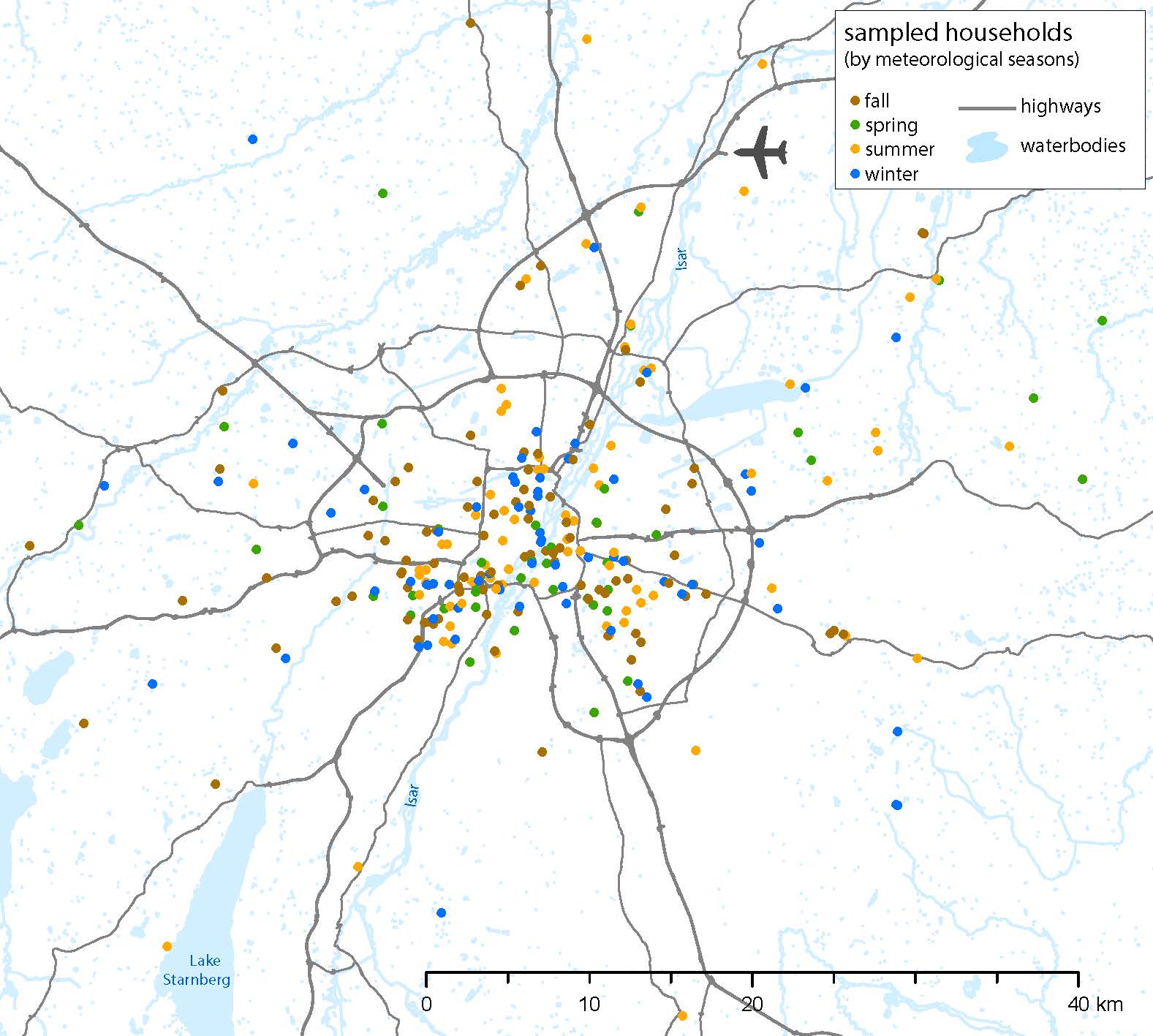


**S1 Files References**

1. Helmholtz-Zentrum München, Epidemiology I: LISAplus. https://www.helmholtz-muenchen.de/en/epi1/research/research-units/research-unit-1-environmental-epidemiology/projects/lisa-plus/index.html (2015). Accessed 17 Nov 2015.

2. Heinrich J, Bolte G, Holscher B, Douwes J, Lehmann I, Fahlbusch B, et al: Allergens and endotoxin on mothers' mattresses and total immunoglobulin E in cord blood of neonates. *The European Respiratory Journal* 2002, **20:**617-623.

3. KOSIS Association Urban Audit: *The German Urban Audit - Comparison of cities in the European Statistical System.* KOSIS Association Urban Audit: Mannheim, Germany. 2013
