# Supplementary material for: Fungal and Bacterial Communities in Indoor Dust Follow Different Environmental Determinants
Source: PLoS One. 2016 Apr 21;11(4):e0154131. doi: 10.1371/journal.pone.0154131 (PMC4839684; doi:10.1371/journal.pone.0154131)
Supplement: S2 File — (DOCX) [file pone.0154131.s002.docx]

S2 File. Environmental **characteristics.**

INDOOR characteristics

For the current investigation we focused on signs of dampness and mould, ventilation habits, type of living room floor, exposure to pets, smoking of tobacco in the flat, number of people living in the home, and renovation measures. All the characteristics were questionnaire-derived.

OUTDOOR characteristics

For questionnaire-based outdoor environmental characteristics, we had information regarding the age of the building, the position of the home (ground floor or higher levels), building density of the neighborhood, whether there were traffic jams around the home during rush hour and if there were facilities with noticeable air pollution near the residency.

Annual average concentrations of particulate matter with an aerodynamic diameter of less than 2.5 μm (PM2.5) and less than 10 μm (PM10), of between 2.5 μm and 10 μm (PMcoarse; coarse particulate matter), PM2.5 absorbance (PM2.5 absorbance, a proxy of black carbon), nitrogen dioxide (NO_2_), and nitrogen oxides NO_2_ and NO (NO_X_) were estimated at the residential addresses at birth by land-use regression (LUR) models developed as part of the European Study of Cohorts for Air Pollution Effects (http://www.escapeproject.eu) (1, 2).

Greenness refers to vegetation level and was defined by the Normalized Difference Vegetation Index (NDVI). Since it is known that plants strongly absorb visible light (from approximately 0.4 µm to 0.7 µm) for use in photosynthesis while strongly reflecting near-infrared light (from 0.7 µm to 1.1 µm) to prevent overheating, NDVI formulae is based on the difference of surface reflectance in these wavelengths (3). In this study, we used cloud-free Landsat 5 TM satellite images at a resolution of 30 m (http://earthexplorer.usgs.gov/), obtained during vegetation rich months (14th of July and 24th of August) to obtain maximum exposure contrasts; NDVI was calculated as the mean value in circular 30m, 100 m and 500 m buffers around the residential address at birth (4, 5).

In addition to air pollution from traffic and greenness, we also considered urbanization grade (urban index) at the place of residence. The urban index was calculated as the proportion of land use with predominantly sealed soil (according to CORINE land cover data, EEA 2006) within a radius of 2 km (6).

| Environmental **characteristics.** | | | n / N^1^ (%) |
| --- | --- | --- | --- |
| **INDOOR environmental characteristic** | |  | |
| **N° of rooms within the flat** (excluding bathroom) | |  | |
| 1-3 rooms | | 51/277 (**18%**) | |
| ≥ 4 rooms | | 226/277 (**82%**) | |
| **N° of occupants in the flat** | |  | |
| 2-3 persons (2 persons: 1% of all households) | | 171/286 (**60%**) | |
| 4 persons | | 91/286 (**32%**) | |
| 5-6 persons (6 persons: 1% of all households) | | 24/286 (**8%**) | |
| **Dampness** | | 18/279 (**6%**) | |
| **Mould at home** | | 102/279 (**37%**) | |
| **Water leakage** | | 34/279 (**12%**) | |
| **Tightness of the windows** (closing with a big or small air gap) | |  | |
| dense | | 207/273 (**76%**) | |
| less dense | | 66/273 (**24%**) | |
| **Ventilation living room through windows - summer** | |  | |
| seldom/never/via another room | | 4/273 (**1%**) | |
| once/several times a day (short) | | 51/273 (**19%**) | |
| once/several times a day (long) | | 148/273 (**80%**) | |
| **Ventilation living room through windows - winter** | |  | |
| seldom/never/via another room | | 15/273 (**5%**) | |
| once/several times a day (short) | | 245/273 (**90%**) | |
| once/several times a day (long) | | 13/273 (**5%**) | |
| **Heating in the home** (no central heating) | | 44/286 (**15%**) | |
| **Renovation measures last 12 months** | | 194/281 (**69%**) | |
| **Pets** (dogs: 4% of all households, cats 6%, birds 5%, others 2%) | | 49/281 (**17%**) | |
| **Type of living room floor** | |  | |
| carpet | | 116/279 (**42%**) | |
| smooth | | 50/279 (**18%**) | |
| smooth with rugs | | 113/279 (**41%**) | |
| **Smoking of tobacco in the flat** | | 35/277 (**13%**) | |
| **OUTDOOR environmental characteristics at birth** | |  | |
| **Age of the building** | |  | |
| Built before 1945 | | 47/277 (**17%**) | |
| Built after 1945 | | 230/277 (**83%**) | |
| **Position of the home** | |  | |
| Ground floor | | 90/279 (**32%**) | |
| 1^st^ floor | | 78/279 (**28%**) | |
| 2^nd^ floor | | 40/279 (**14%**) | |
| 3^rd^ floor or higher | | 71/279 (**25%**) | |
| **Residential density of the neighborhood** | |  | |
| high | 115/273 (**42%**) | | |
| average | 141/273 (**52%**) | | |
| low | 17/273 (**6%**) | | |
| **Traffic jams in rush hour** | 36/273 (**13%**) | | |
| **Surrounding greenness (500-m buffer), median (p25%-p75%)** | 0.32 (0.27-0.37) 281 | | |
| **Surrounding greenness (100-m buffer), median (p25%-p75%)** | 0.30 (0.24-0.35) 281 | | |
| **Surrounding greenness (30-m buffer), median (p25%-p75%)** | 0.29 (0.22-0.36) 281 | | |
| **Urban index** |  | | |
| Urban (≥ 0.7) | 140/281 (**50%**) | | |
| Semi-urban (> 0.3 and < 0.7) | 80/281 (**28%**) | | |
| Rural (≤ 0.3) | 61/281 (**22%**) | | |
| **NO_2_** µg/m^3^, **median (p25%-p75%)** | 21.78 (17.59-26.71) 281 | | |
| **NO_x_** µg/m^3^, **median (p25%-p75%)** | 35.66 (29.55-42.99) 281 | | |
| **PM_2.5_** µg/m^3^, **median (p25%-p75%)** | 13.42 (12.8-14.19) 281 | | |
| **PM_10_** µg/m^3^, **median (p25%-p75%)** | 20.47 (19.07-21.92) 281 | | |
| **PM_coarse_** µg/m^3^, **median) (p25%-p75%)** | 6.58 (5.66-7.85) 281 | | |
| **PM absorbance** 10^-5^ *****µg/m^3^, **median (p25%-p75%)** | 1.68 (1.55-1.8) 281 | | |
| **Facility with noticeable air pollution nearby** (between 50 and 100 m) | 21/277 (**8%**) | | |
| **Facility with noticeable air pollution nearby** (within 50 m) | 12/277 (**4%**) | | |
| **Season of dust sampling** (sampling date) |  | | |
| Winter | 64 / 286 (**22%**) | | |
| Spring | 45 / 286 (**16%**) | | |
| Summer | 76 / 286 (**27%**) | | |
| Autumn | | 101 / 286 (**35%**) | |
| ^1^For most variables, data were not available for all samples, due to errors in questionnaires or measurements. N in the table represents samples with fungal fingerprints, N for bacterial fingerprints differed by 1–3 samples (286→283, 281→278, 277→274, 279→276, 273→274, or 188→189, respectively) | | | |

**S2 File references**

1. Cyrys J, M. Eeftens, J. Heinrich, C. Ampe, A. Armengaud, R. Beelen , T. Bellander, T. Beregszaszi, M. Birk, G. Cesaroni, M. Cirach, K. de Hoogh, A. De Nazelle, F. de Vocht, C. Declercq, A. Dédelé, K. Dimakopoulou, K. Eriksen, C. Galassi, R. Gražulevičiené, G. Grivas, O. Gruzieva, A. Hagenbjörk Gustafsson, B. Hoffmann, M. Iakovides, A. Ineichen, U. Krämer, T. Lanki, P. Lozano, C. Madsen, K. Meliefste, L. Modig, A. Mölter, G. Mosler, M. Nieuwenhuijsen, M. Nonnemacher, M. Oldenwening, A. Peters, S. Pontet, N. Probst-Hensch, U. Quass, O. Raaschou-Nielsen, A. Ranzi, D. Sugiri, E. G. Stephanou, P. Taimisto, M.-Y. Tsai, É. Vaskövi, S. Villani, M. Wang, B. Brunekreef, G. Hoek. Variation of NO_2_ and NO concentrations between and within 38 European study areas: results from the ESCPAE study. Atmos Environ. 2012;62:374-90.

2. Eeftens M, M-Y. Tsai, C, Ampe, B. Anwander, R. Beelen, T. Bellander, G. Cesaroni, M. Cirach, J. Cyrys, K. de Hoogh, A. De Nazelle, F. de Vocht, C. Declercq, A. Dédelé, K. Eriksen, C. Galassi, R. Gražulevičiené, G. Grivas, J. Heinrich, B. Hoffmann, M. Iakovides, A. Ineichen, K. Katsouyanni, M. Korek, U. Krämer, T. Kuhlbusch, T. Lanki, C. Madsen, K. Meliefste, A. Mölter, G. Mosler, M. Nieuwenhuijsen, M. Oldenwening, A. Pennanen, N. Probst-Hensch, U. Quass, O. Raaschou-Nielsen, A. Ranzi, E. Stephanou, D. Sugiri, O. Udvardy, É. Vaskövi, G. Weinmayr, B. Brunekreef, G. Hoek. Variation of PM2.5, PM10, PM2.5 absorbance and PMcoarse concentrations between and within 20 European study areas – results of the ESCAPE project. Atmos Environ. 2012;62:303-17.

3. Weier J, Herring D. Measuring Vegetation (NDVI & EVI). 2000. [http://earthobservatory.nasa.gov/Features/MeasuringVegetation/]

4. Markevych I, Thiering E, Fuertes E, Sugiri D, Berdel D, Koletzko S, et al. A cross-sectional analysis of the effects of residential greenness on blood pressure in 10-year old children: results from the GINIplus and LISAplus studies. BMC Public Health. 2014;14:477.

5. Markevych I, Fuertes E, Tiesler CM, Birk M, Bauer CP, Koletzko S, et al. Surrounding greenness and birth weight: results from the GINIplus and LISAplus birth cohorts in Munich. Health & place. 2014;26:39-46.

6. Jochner SC, Sparks TH, Estrella N, Menzel A. The influence of altitude and urbanisation on trends and mean dates in phenology (1980-2009). Int J Biometeorol. 2012;56(2):387-94.
