# Supplementary material for: Fungal and Bacterial Communities in Indoor Dust Follow Different Environmental Determinants
Source: PLoS One. 2016 Apr 21;11(4):e0154131. doi: 10.1371/journal.pone.0154131 (PMC4839684; doi:10.1371/journal.pone.0154131)
Supplement: S1 Table — (DOCX) [file pone.0154131.s003.docx]

**S1 Table. Community variation.** Significance of associations between environmental determinants and microbiome community variation (Adonis *P*, MRPP *δ*) with respective *R^2^* (Adonis) and *A* (MRPP chance corrected between groups agreement) values which are not shown in the paper itself; bold: *P* or *δ* ≤ 0.05

|  | Fungi |  |  |  | Bacteria |  |  |  |
| --- | --- | --- | --- | --- | --- | --- | --- | --- |
| **Environmental characteristics** | *P* | *R^2^* | *δ* | *A* | *P* | *R^2^* | *δ* | *A* |
| N° of rooms within the flat | 0.75 | 0.001 | 0.87 | < 0.001 | 0.71 | 0.001 | 0.79 | < 0.001 |
| N° of occupants in the flat | 0.85 | 0.003 | 0.79 | < 0.001 | 0.36 | 0.008 | 0.28 | 0.001 |
| N° of infants in the flat | 0.90 | 0.002 | 0.86 | < 0.001 | 0.45 | 0.006 | 0.38 | < 0.001 |
| Gender of the youngest infant | 0.26 | 0.004 | 0.30 | < 0.001 | 0.31 | 0.004 | 0.49 | < 0.001 |
| Dampness | 0.69 | 0.002 | 0.29 | < 0.001 | 0.16 | 0.007 | 0.39 | < 0.001 |
| Mould at home | **0.04** | 0.012 | **0.03** | 0.004 | 0.09 | 0.009 | 0.13 | 0.002 |
| Water leakage | 0.81 | 0.001 | 0.85 | < 0.001 | 0.57 | 0.002 | 0.62 | < 0.001 |
| Tightness of the windows | **0.03** | 0.013 | **0.04** | 0.004 | 0.36 | 0.004 | 0.36 | < 0.001 |
| Ventilation living room through windows - summer | 0.27 | 0.009 | 0.24 | 0.001 | 0.71 | 0.004 | 0.93 | < 0.001 |
| Ventilation living room through windows - winter | 0.67 | 0.005 | 0.64 | < 0.001 | **0.05** | 0.017 | **0.05** | 0.007 |
| Heating within the home | **0.03** | 0.013 | **0.02** | 0.005 | 0.36 | 0.004 | 0.41 | < 0.001 |
| Renovation measures last 12 months | 0.44 | 0.003 | 0.61 | < 0.001 | 0.65 | 0.002 | 0.65 | < 0.001 |
| Pets | 0.27 | 0.004 | 0.28 | < 0.001 | 0.62 | 0.002 | 0.75 | < 0.001 |
| Type of living room floor | **< 0.001** | 0.057 | **< 0.001** | 0.023 | 0.08 | 0.015 | **0.02** | 0.009 |
| Smoking of tobacco in the flat | 0.42 | 0.003 | 0.41 | < 0.001 | 0.71 | 0.001 | 0.78 | < 0.001 |
| Age of the building | **0.01** | 0.017 | **0.01** | 0.005 | 0.28 | 0.005 | 0.31 | < 0.001 |
| Position of the home | 0.49 | 0.010 | 0.67 | < 0.001 | 0.10 | 0.015 | **0.05** | 0.007 |
| Building density of the neighborhood | 0.52 | 0.006 | 0.59 | < 0.001 | 0.39 | 0.007 | 0.44 | < 0.001 |
| Traffic jams in rush hour | 0.83 | 0.001 | 0.83 | < 0.001 | 0.29 | 0.004 | 0.24 | < 0.001 |
| Facility with noticeable air poll. within 50 and 100 m | 0.58 | 0.002 | 0.72 | < 0.001 | 0.45 | 0.003 | 0.40 | < 0.001 |
| Facility with noticeable air pollution within 50 m | 0.33 | 0.004 | 0.28 | < 0.001 | 0.85 | < 0.001 | 0.94 | < 0.001 |
| Surrounding greenness (500 m buffer) | 0.72 | 0.004 | 0.63 | < 0.001 | 0.84 | 0.003 | 0.94 | < 0.001 |
| Surrounding greenness (100 m buffer) | **0.05** | 0.017 | **0.006** | 0.008 | 0.19 | 0.011 | 0.22 | 0.002 |
| Surrounding greenness (30 m buffer) | 0.06 | 0.016 | **0.01** | 0.007 | 0.33 | 0.008 | 0.30 | < 0.001 |
| Urban index | **0.02** | 0.021 | **0.01** | 0.007 | 0.51 | 0.006 | 0.60 | < 0.001 |
| NO_2_ | 0.23 | 0.010 | 0.06 | 0.004 | 0.63 | 0.005 | 0.75 | < 0.001 |
| NO_x_ | 0.06 | 0.016 | **0.03** | 0.005 | 0.37 | 0.008 | 0.41 | < 0.001 |
| PM_2.5_ | **0.004** | 0.028 | **0.005** | 0.009 | 0.51 | 0.006 | 0.44 | < 0.001 |
| PM_10_ | 0.54 | 0.006 | 0.32 | < 0.001 | 0.82 | 0.003 | 0.70 | < 0.001 |
| PM_coarse_ | **0.04** | 0.018 | **0.008** | 0.008 | 0.41 | 0.007 | 0.46 | < 0.001 |
| PM**_2.5_** absorbance | 0.07 | 0.015 | **0.06** | 0.004 | 0.37 | 0.008 | 0.42 | < 0.001 |
