# Supplementary material for: Fungal and Bacterial Communities in Indoor Dust Follow Different Environmental Determinants
Source: PLoS One. 2016 Apr 21;11(4):e0154131. doi: 10.1371/journal.pone.0154131 (PMC4839684; doi:10.1371/journal.pone.0154131)
Supplement: S2 Table — (DOCX) [file pone.0154131.s004.docx]

S2 Table. Significance of associations with diversity – all variables. Significance of associations between all environmental determinants and microbial diversity (Simpson and Shannon indices, *P*-values (Wilcoxon signed rank test or Kruskall–Wallis test)); bold: *P* ≤ 0.05

|  | Fungi |  | Bacteria |  |
| --- | --- | --- | --- | --- |
| **Environmental characteristics** | *P* (Simpson 1-D) | *P* (Shannon H’) | *P* (Simpson 1-D) | *P* (Shannon H’) |
| N° of rooms within the flat | 0.13 | 0.13 | 0.69 | 0.50 |
| N° of occupants in the flat | 0.88 | 0.80 | **0.03** | **0.01** |
| Dampness | 0.10 | 0.12 | 0.17 | 0.23 |
| Mould at home | 0.88 | 0.71 | 0.77 | 0.57 |
| Water leakage | 0.60 | 0.78 | 0.27 | 0.17 |
| Tightness of the windows | 0.78 | 0.87 | 0.78 | 0.85 |
| Ventilation living room through windows - summer | 0.08 | **0.04** | 0.62 | 0.56 |
| Ventilation living room through windows - winter | 0.49 | 0.32 | 0.13 | 0.20 |
| Heating within the home | 0.30 | 0.31 | 0.77 | 0.91 |
| Renovation measures last 12 months | 0.69 | 0.71 | 0.75 | 0.52 |
| Pets | 0.84 | 0.74 | 0.55 | 0.91 |
| Type of living room floor | 0.30 | 0.33 | **0.04** | **0.01** |
| Smoking of tobacco in the flat | 0.13 | 0.12 | 0.75 | 0.80 |
| Age of the building | 0.31 | 0.49 | 0.24 | 0.36 |
| Position of the home | 0.15 | 0.09 | **0.04** | **0.03** |
| Building density of the neighborhood | 0.46 | 0.27 | 0.67 | 0.62 |
| Traffic jams in rush hour | 0.99 | 0.59 | 0.53 | 0.44 |
| No facility with noticeable air pollution nearby | 0.32 | 0.43 | 0.44 | 0.54 |
| Facility with noticeable air poll. within 50 and 100 m | 0.17 | 0.25 | 0.26 | 0.27 |
| Facility with noticeable air pollution within 50 m | 0.16 | 0.11 | 0.41 | 0.40 |
| Surrounding greenness (500 m buffer) | 0.09 | 0.14 | 0.69 | 0.69 |
| Surrounding greenness (100 m buffer) | **0.03** | 0.11 | 0.14 | 0.11 |
| Surrounding greenness (30 m buffer) | 0.32 | 0.31 | 0.59 | 0.56 |
| Urban index | 0.37 | 0.44 | 0.65 | 0.73 |
| NO_2_ | 0.25 | 0.35 | 0.54 | 0.51 |
| NO_x_ | 0.14 | 0.33 | 0.43 | 0.29 |
| PM_2.5_ | 0.72 | 0.60 | 0.74 | 0.81 |
| PM_10_ | 0.35 | 0.32 | 0.91 | 0.85 |
| PM_coarse_ | 0.10 | 0.15 | 0.62 | 0.66 |
| PM_2.5_ absorbance | 0.66 | 0.49 | 0.62 | 0.75 |
